# Breath Analysis Using eNose and Ion Mobility Technology to Diagnose Inflammatory Bowel Disease—A Pilot Study

**DOI:** 10.3390/bios9020055

**Published:** 2019-04-12

**Authors:** Akira Tiele, Alfian Wicaksono, Jiten Kansara, Ramesh P. Arasaradnam, James A. Covington

**Affiliations:** 1 School of Engineering, University of Warwick, Coventry CV4 7AL, UK; F-A.Tiele@warwick.ac.uk (A.T.); A.Wicaksono@warwick.ac.uk (A.W.); 2 Department of Gastroenterology, University Hospitals Coventry and Warwickshire, Coventry CV2 2DX, UK; Jiten.Kansara@uhcw.nhs.uk (J.K.); R.Arasaradnam@warwick.ac.uk (R.P.A.); 3 Applied Biological Sciences, Coventry University, Coventry CV1 5FB, UK; 4 Health and Life Sciences, University of Leicester, Leicester LE1 7RH, UK; 5 Warwick Medical School, University of Warwick, Coventry CV4 7AL, UK

**Keywords:** inflammatory bowel disease (IBD), breath analysis, volatile organic compounds (VOCs), faecal calprotectin (FCP), electronic nose (eNose), GC-IMS

## Abstract

Early diagnosis of inflammatory bowel disease (IBD), including Crohn’s disease (CD) and ulcerative colitis (UC), remains a clinical challenge with current tests being invasive and costly. The analysis of volatile organic compounds (VOCs) in exhaled breath and biomarkers in stool (faecal calprotectin (FCP)) show increasing potential as non-invasive diagnostic tools. The aim of this pilot study is to evaluate the efficacy of breath analysis and determine if FCP can be used as an additional non-invasive parameter to supplement breath results, for the diagnosis of IBD. Thirty-nine subjects were recruited (14 CD, 16 UC, 9 controls). Breath samples were analysed using an in-house built electronic nose (Wolf eNose) and commercial gas chromatograph–ion mobility spectrometer (G.A.S. BreathSpec GC-IMS). Both technologies could consistently separate IBD and controls [AUC ± 95%, sensitivity, specificity], eNose: [0.81, 0.67, 0.89]; GC-IMS: [0.93, 0.87, 0.89]. Furthermore, we could separate CD from UC, eNose: [0.88, 0.71, 0.88]; GC-IMS: [0.71, 0.86, 0.62]. Including FCP did not improve distinction between CD vs. UC; eNose: [0.74, 1.00, 0.56], but rather, improved separation of CD vs. controls and UC vs. controls; eNose: [0.77, 0.55, 1.00] and [0.72, 0.89, 0.67] without FCP, [0.81, 0.73, 0.78] and [0.90, 1.00, 0.78] with FCP, respectively. These results confirm the utility of breath analysis to distinguish between IBD-related diagnostic groups. FCP does not add significant diagnostic value to breath analysis within this study.

## 1. Introduction

Inflammatory bowel disease (IBD) is a chronic condition of unknown aetiology, which includes Crohn’s disease (CD) and ulcerative colitis (UC) [1]. Both conditions involve inflammation of the gut and are particularly unpleasant. While UC only affects the colon (large intestine), CD can affect any part of the digestive system from the mouth to the anus [2]. IBD is a common condition in the Western world, affecting over 250,000 people in the UK and 28 million worldwide [3]. The estimated annual cost of treatment, per patient, is approx. €30,000 with an average of 20% loss of working productivity [1,4]. This is due to the relapsing nature of the disease—there may be times when the symptoms are severe (flare-ups), followed by long periods when there are few or no symptoms at all (remission). Common symptoms include diarrhoea, cramping pains in the abdomen, vomiting, weight-loss and fatigue [5]. In addition to this, IBD has a damaging impact on psychosocial functioning, quality of life, and significant personal cost of delayed treatment [6]. A study from 2014, conducted in the UK, revealed that 10% of IBD patients are initially misdiagnosed with other gastrointestinal conditions, such as irritable bowel syndrome (IBS), and that 3% of misdiagnosed cases persisted for five or more years [7]. Misdiagnosis can have serious consequences for the patient; especially for those with CD, since delays are correlated with an increased risk of later bowel stenosis and CD-related intestinal surgery [7]. Early diagnosis of IBD remains a clinical challenge, with current tests being invasive and costly. Diagnostic tools for CD and UC include a thorough history, endoscopic investigations with histological examination, faecal inflammatory markers, capsule endoscopy and imaging [8]. Colonoscopy with histology is considered the “gold standard” to diagnose IBD [9]. This procedure is uncomfortable for the patient, often involves multiple biopsies, is expensive for the health service provider (such as the NHS) and has an associated morbidity.

In recent years, breath and stool analysis have shown increasing potential as non-invasive diagnostic tools. This includes the analysis of exhaled volatile organic compounds (VOCs) and biomarkers of inflammation. There are an estimate of over 3000 VOCs in human breath [10], which are a combination of bi-products of normal metabolic activity and, in some cases, specific biomarkers associated with a disease [11,12,13,14]. In a review paper from 2018, A. Wilson [15] investigated the increasing application of electronic nose (eNose) technology for the clinical diagnosis of gastrointestinal diseases, including IBD, bile acid diarrhoea, colorectal cancer (CRC), IBS, and others. A number of these studies explored IBD utilising breath-based methods, employing analytical technologies such as selected ion flow tube mass spectrometry (SIFT-MS) [16], gas chromatography time-of-flight mass spectrometry (GC-TOF-MS) [17] and ion molecule reaction mass spectrometry (IMR-MS) [18]. These studies demonstrated impressive efficacy and performance results, suggesting a strong link between exhaled VOCs and IBD. It has been proposed that these VOCs are generated from microbe-associated gases, which originate in the gut and diffuse into the bloodstream and enter the lung’s alveoli, where they are eventually expelled [19]. Moreover, CD and UC have both shown distinct patterns of VOCs, reflecting gut fermentome metabolites [8,20].

In the domain of measurable objective biomarkers, C-reactive protein (CRP) and faecal calprotectin (FCP) have been gaining clinical research interest. CRP correlates reasonably well with CD activity, but has poor sensitivity [21]. Moreover, it is found in blood plasma and cannot be monitored non-invasively. In contrast, FPC correlates well with both CD and UC and can be monitored in stool. Stool samples are routinely collected to inform the management of many gastrointestinal diseases and infections [22]; however, patient compliance is rarely >60%, due to embarrassment or concerns about results [23]. Nonetheless, FCP presents an additional non-invasive parameter, which may compliment results from breath testing.

The aim of this pilot study is to evaluate the efficacy of breath analysis and determine if FCP can be used to supplement breath results for the diagnosis of IBD. The first objective of this study is to determine whether exhaled VOCs can be used to distinguish IBD from healthy controls and CD from UC, using a commercial gas chromatograph—ion mobility spectrometer (GC-IMS) and an in-house built electronic nose (eNose). To the best of our knowledge, this breath study is the first to investigate CD, UC and healthy controls using GC-IMS and eNose technology. The key advantages of these technologies are that they are non-invasive, portable, relatively inexpensive, applicable for high-throughputs and have sampling procedures suitable for nearly all patient demographics, including vulnerable subjects such as children and the elderly. Confounding factors, such as BMI, smoking habits and gender will be used to verify analysis efficacy. The second objective of this study is to determine whether FCP, used as an additional non-invasive parameter, adds diagnostic value to breath analysis in IBD.

## 2. Materials and Methods

### 2.1. Subjects

A total of 39 subjects were recruited for this pilot study, as part of the larger ‘Famished’ study. Ethical approval was obtained from the Warwickshire research ethics committee (IRAS ref: 18717). 30 patients had a histologically confirmed IBD (14 CD, 16 UC), as well as 9 healthy control volunteers. IBD patients were recruited from dedicated IBD clinics at University Hospitals Coventry and Warwickshire (UHCW), UK. Details of medication and disease activity were recorded and simple colitis activity index (SCAI) for UC and Harvey Bradshaw index (HBI) for CD were calculated at the time of recruitment. Healthy controls were volunteers who did not report any overt gastrointestinal symptoms and were not on routine oral medication or recovering from any recent illnesses. An overview of the demographic data of IBD patients and healthy controls is shown in Table 1. The mean age of the IBD cohort was 49.7 years (standard deviation 17.5) and there were 18 males and 12 females.

**Figure 1:**
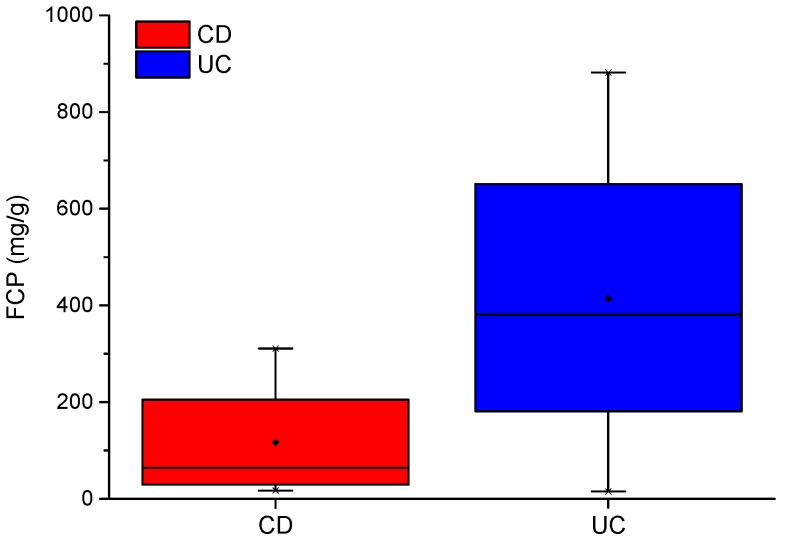
CD and UC boxplots for FCP scores.

As shown in Table 1, inflammation parameters such as CRP and FCP were recorded for the IBD cohort. FCP is a good indicator of inflammation in the bowel [24]. FCP levels between CD and UC patients have been shown to differ by over 55 ug/g (higher in those with UC) [25]. In our IBD cohort, mean FCP between CD and UC patients differ by almost 300 ug/g. Mean scores of 117 and 414 ug/g, respectively, indicate that the CD group is in remission and the UC group has active disease. A box plot of FCP scores for CD and UC are shown in Figure 1.

### 2.2. Electronic Nose (eNose)

The term ‘eNose’ describes an instrument formed from an array of sensors with overlapping sensitivity [26]. Electronic noses are used in food and drink-related industries [27], as well as environmental monitoring [28]. Recent advancements in eNose technologies, such as improvements in gas-sensor design and pattern-recognition algorithms, have led to increased applicability of eNoses in the medical domain [29]. The Wolf eNose system (Warwick OLFaction: Wolf) was built in-house at the School of Engineering, University of Warwick [30]. The device consists of 13 sensors, employing a range of different sensor technologies, including eight amperometric electro-chemical sensors (Alphasense Ltd., Essex, UK), two non-dispersive infra-red (NDIR) optical devices (Clairair Ltd., Essex, UK) and a single photo-ionisation detector (Mocon, Minneapolis, USA). The sensors deployed in the Wolf eNose are summarised in Table 2.

Since the Wolf eNose was designed and developed in-house, there is a high level of freedom allowing the instrument to be tailored to specific applications. A custom sample injection system was developed for this study, to enhance the capabilities of the Wolf eNose to analyse breath samples.

**Figure 2:**
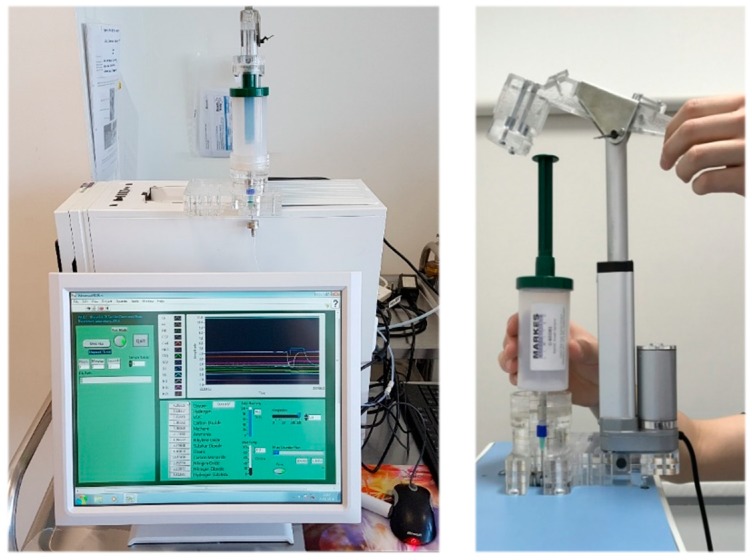
Wolf eNose and custom Bio-VOC injection system.

Alveolar breath samples, for Wolf eNose analysis, were collected using a commercially-available breath sampling device, known as the Bio-VOC (Markes Int., Llantrisant, UK). Alveolar breath refers to the last portion (350 mL) of exhaled breath, expelled from within the lungs and the lower-airways, which have undergone gaseous exchange with the blood in the alveoli [31]. A healthy adult expires approximately 500 mL air with each breath, of which the first 150 mL consist of dead-space air (no transfer of oxygen) from the upper-air ways and nasopharynx [32]. Subjects were asked to perform a single slow vital capacity breath into a Bio-VOC unit, in order to trap the last 129 mL of exhaled breath [33]. Subjects were supplied with a disposable carboard mouthpiece, and the Bio-VOC was cleaned thoroughly using antibacterial, alcohol-free sanitary wipes after every sample. Collected breath samples were injected into the Wolf eNose inlet port using a custom linear-actuator injection system. The injection system was manufactured using 5 mm acrylic sheets to support a 12 V, 200 mm linear actuator motor (JS-TGZ, Jianshun, Shenzhen, China). The Bio-VOC was secured into the structure, as shown in Figure 2. Thereafter, the plunger was compressed automatically, at a constant rate, over a 30-s injection period.

**Figure 3:**
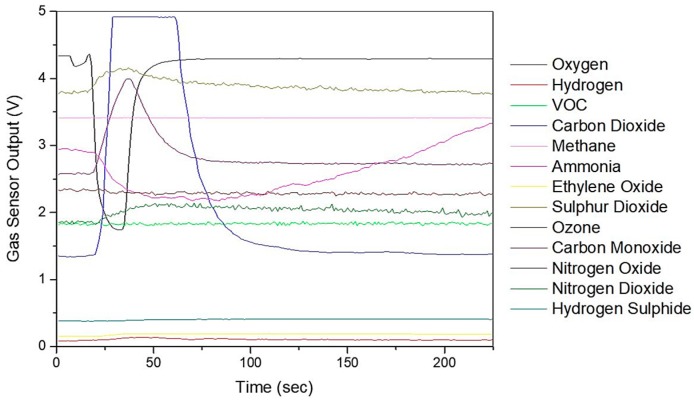
Wolf eNose typical sensor output responses.

Figure 3 shows the typical sensors responses from the Wolf eNose to the exhaled breath sample of a CD patient. The chemicals refer to the target the sensor is sold to sense.

### 2.3. Gas Chromatography—Ion Mobility Spectrometry (GC-IMS)

A more recent, alternative approach to eNoses has been the use of portable GC-IMS analysers, which have demonstrated capabilities in medical diagnostics [34]. The BreathSpec GC-IMS (G.A.S., Dortmund, Germany) is a commercially-available instrument, consisting of a gas chromatograph (GC) and an ion mobility spectrometer (IMS), collectively known as GC-IMS. This instrument uses a GC as a pre-separator (based on chemical interactions with the column), followed by a IMS detector. In this case, the BreathSpec is equipped with a SE54 mid-range polarity column. The IMS uses a drift tube where the time taken for molecules to traverse the tube against a buffer gas (in this case nitrogen) is measured. This buffer gas is generated using a Nitrostation 50LC (Leman Instruments, Geneva, Switzerland) with 99.999% purity. The gas slows down the ions resulting in larger ions being slowed more than smaller ones. Ions are then collected on the detector (Faraday plate), to deliver a time-dependent signal that corresponds with ion mobility [35]. This technique can measure substances in the low parts-per-billion (ppb) range and delivers measurement results in less than 10 min.

**Figure 4:**
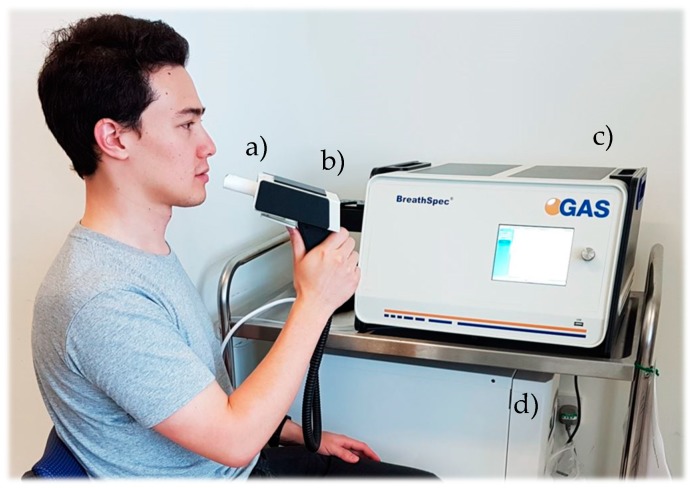
G.A.S. BreathSpec GC-IMS: (**a**) Plastic disposable mouthpiece; (**b**) Mouthpiece holder; (**c**) GC-IMS instrument; (**d**) Nitrogen generator.

The Bio-VOC was not required for G.A.S. BreathSpec GC-IMS analysis. Subjects were provided with a disposable plastic mouthpiece, which pushes into the mouthpiece holder/sample inlet and connects directly to the side-panel of the instrument. This sampling procedure also collects end-tidal breath, since only the last four seconds of exhaled breath are collected for analysis [36]. The G.A.S. BreathSpec GC-IMS instrument is shown in Figure 4.

**Figure 5:**
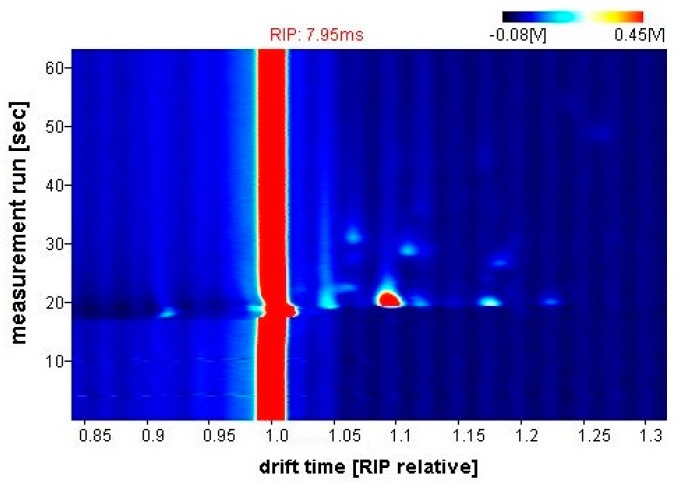
G.A.S. BreathSpec GC-IMS output example (CD patient).

Figure 5 shows a typical output response (chromatogram) from the G.A.S. BreathSpec GC-IMS. The obtained sample is represented as a topographic map, whereby each datapoint is characterised by the retention time in the chromatographic column (seconds), the drift time (milliseconds) and the intensity of ion current signal (millivolts), indicated by colour. Laboratory Analytical Viewer (LAV) software (v2.2.1, G.A.S., Dortmund, Germany) was used to analyse the chromatograms.

### 2.4. Data Analysis

Both the Wolf eNose and G.A.S. BreathSpec GC-IMS require an initial feature extraction step before classification. The aim of feature extraction is to select robust information from the characteristic response; for example, selecting the maximum value of the original sensor response curve [37]. Supervised feature selection and class prediction was performed using a *k*-fold cross-validation method, where *k* = 10. This method involves partitioning the original data set into 10 equally-sized subsets. Of the 10 subsets, a single subset is retained as the validation data for testing the model, and remaining subsets are used as training data. This process is repeated 10 times (number of folds), with each subset used once as validation data. The 10 results are then combined to produce a single estimation. This method for cross-validation is commonly used in breath analysis, to ensure robustness and avoid ‘false-negative’ errors (also known as type II errors) [38]. These occur when a test result indicates that a condition is true, when it is known to be false [39]. In our case, this would refer to a test result that indicates that a subject has IBD, when they are in fact from the healthy control group. A Wilcoxon rank sum test was used to calculate p-values for each feature, with the most informative features used for classification. This is undertaken inside the fold, to remove potential over fitting. Class predictions and sensitivity/specificity calculations were performed using five classification algorithms, specifically: support vector machine (SVM), sparse logistic regression (SLR), Gaussian process, neural network, and random forest (RF).

In addition to this analysis, the G.A.S. BreathSpec GC-IMS can potentially identify unknown VOCs that contribute significantly to the classification analysis. Using GC-IMS Library Search software (v1.0.1, G.A.S., Dortmund, Germany), we can identify compounds based on gas chromatographic retention times and ion mobility drift times, by referring to a NIST database. The database includes about 400,000 annotated retention indices and an estimated 83,000 compound entries [40]. To identify unknown compounds, GC-IMS files were loaded into the GCxIMS software and VOC identification is performed by simply clicking on the region of interest. The software then refers to the NIST database to generate a list of likely compound matches. A retention time range is provided, to indicate whether the suggested compound matches the expected retention time on the topographic map. Compounds with a close match in retention time and chemical structure were chosen as the identified compound.

### 2.5. Quality Assurance and Control

For quality assurance, the position and quality of the reactive ion peak (RIP) on the GC-IMS was regularly checked for signs of contamination. The RIP refers to the constant peak in the spectrum, which results from the carrier gas being always present in the measurement process. Moreover, samples were collected in the same setting, by the same operator, throughout the entire study. This is an important factor to ensure consistent sampling procedures since the collection process is manually triggered by the operator, while the subject exhales through the mouthpiece. Furthermore, the GC-IMS instrument was normalised using a standard ketone mix (2-butanone, 2-pentanone, 2-hexanone, 2-heptanone, 2-octanone and 2-nonanone), to match the GC-IMS Library Search software with the equipped column. For Wolf eNose calibration, the headspace gas from several chemical standards were tested (ketones, esters, alcohols, alkanes and aromatics). These experiments revealed various relationships between sensor responses and concentrations. Furthermore, testing the different compounds individually produced responses from different sets of sensors, which confirms a degree of selectivity [30]. In addition to this, quality control procedures were implemented. This involved collecting regular room air samples to monitor changes in ambient air and identify possible exogenous VOCs, i.e., compounds which do not originate from within the body.

### 2.6. Confounding Factors

In a recent study, Blanchet et al. [41] explored factors that influence the VOC content in human breath and stated that any application of exhaled air for diagnostics should consider possible confounders. For this IBD study, the following confounders were considered: body mass index (BMI), smoking habits and gender. BMI categories include: underweight (<18.5 kg/m^2^), normal weight (18.5–24.9 kg/m^2^), overweight (25.0–29.9 kg/m^2^) and obese (>30.0 kg/m^2^) [42]. To simplify the analysis, underweight and normal weight were combined into a single category, as well as overweight and obese. Smokers can be broadly defined as individuals who have smoked at least 100 cigarettes in their lifetime [43]. Thus, never smokers are defined as adults who have never smoked or have smoked less than 100 cigarettes in their lifetime. These definitions were used to categorise smokers and non-smokers. Gender groups were divided into male and female—this factor is of particular importance, since it was not possible to attain a gender balanced UC group during recruitment. The confounding factor groups are shown in Table 3, with roughly evenly matched groups and various combinations of CD, UC and control subjects.

## 3. Results

**Figure 6:**
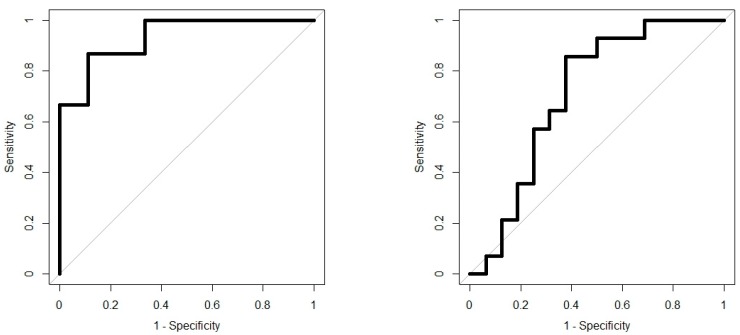
IBD analysis: G.A.S. BreathSpec GC-IMS ROC curves; IBD vs. Controls (**left**); CD vs. UC (**right**).

**Figure 7:**
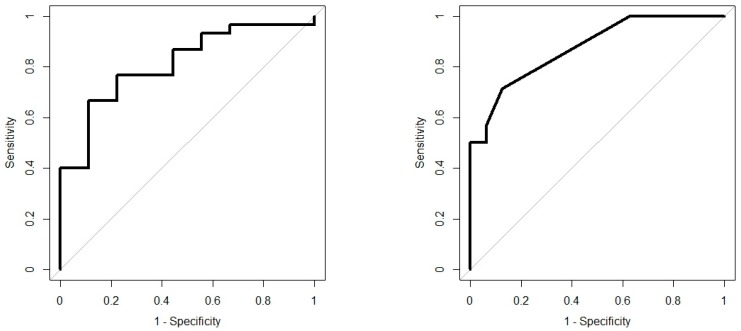
IBD analysis: Wolf eNose ROC curves; IBD vs. Controls (**left**); CD vs. UC (**right**).

Analysis results are presented as operating characteristic (ROC) curves. The associated area under curve (AUC) is a measure of how well parameters can distinguish between diagnostic groups. In our case, the groups were IBD vs. controls, and CD vs. UC. Generated ROC curves for G.A.S. BreathSpec GC-IMS and Wolf eNose, IBD vs. controls and CD vs. UC, are shown in Figure 6 and Figure 7, respectively. The RF algorithm consistently performed best. The analysis results are summarised in Table 4 and Table 5.

### 3.1. Chemical Identification

VOC analysis indicates that two compounds play a crucial role in distinguishing between IBD and controls. Chemical identification for the BreathSpec instrument, using the GC-IMS Library Search software, suggests that the best matches for the identified compounds include: butanoic acid (2-methyl-, propyl ester) and ethanoic acid (3-methyl-1-butyl ester).

**Figure 8:**
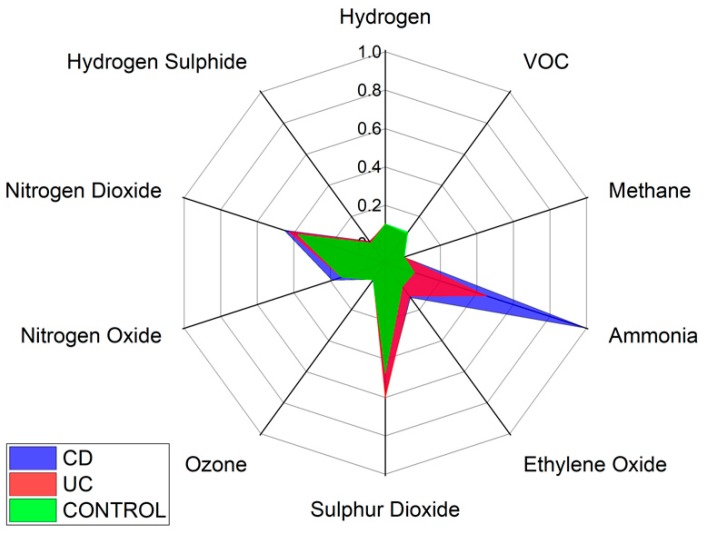
Wolf eNose sensor responses radar plot.

Specific VOCs cannot be identified using the Wolf eNose. However, in an attempt to identify the chemical groups, which contribute most to the seperation between diagnostic groups, we have analysed the normalised average change in Wolf eNose outputs, per group. A radar plot of the responses is shown in Figure 8.

Ammonia, sulphur dioxide (SO_2_) and nitrogen dioxide (NO_2_) sensors showed the greatest changes in sensor outputs and thereby contributed most to the separation between diagnostic groups. Significant changes were observed in ammonia by both UC and CD patients. Changes in NO_2_ were marginally greater in CD over UC and controls, while UC is associated with increased sulphur dioxide. The other sensor outputs show small variations between groups, such as increased ethylene oxide in UC over controls.

### 3.2. Confounding Factors

The analysis previously conducted on IBD and control groups was repeated, using the same analytical techniques and algorithms, on the confounding factor groups, i.e., BMI: under- & normal weight vs. overweight & obese; smoking: smokers vs. never smokers; gender: male vs. female. The analysis results are summarised in Table 6 and Table 7. 

Table 6 and Table 7 demonstrate that the possible confounding factors of BMI, smoking and gender have insignificant influence on breath content. This is particularly true for BMI and smoking, as they achieve an AUC of around 50 for both technologies. Gender seems to have the most influence on breath content, with an AUC of around 60.

### 3.3. Faecal Calprotectin (FCP)

The demonstrated Wolf eNose analysis for CD vs. UC was repeated using FCP as an additional feature, on a reduced dataset of 20 samples (11 CD, 9 UC) to match the availability of FCP scores. This analysis could not be repeated on the G.A.S. BreathSpec GC-IMS, because the features from this device are made up of clusters with numerous data points and are thus not directly compatible with single feature values, such as FCP.

The combined breath with FCP analysis was compared to breath without FCP, for the same dataset. The analysis results are summarised in Table 8. In addition to this, we investigated whether the combined analysis of breath with FCP could better distinguish between CD vs. controls and UC vs. controls, as shown in Table 9.

## 4. Discussion

In recent years, numerous studies have investigating the efficacy of breath VOCs to diagnose IBD [16,44,45]. At least three studies utilised selected ion flow tube mass spectrometry (SIFT-MS) to distinguish IBD from healthy controls, as well as separating UC from CD. Hicks et al. [44] identified 6 VOCs (hydrogen cyanide, ammonia, dimethyl sulphide, hydrogen sulphide, butanal, and nonanal) which significantly differed in concentrations, between diagnostic groups. Another study identified 3 specific VOCs (1-octene, 1-decene, (E)-2-nonene) as relevant for predicting the presence of IBD (AUC 0.96), but did not identify any significant difference in VOCs between CD and UC [16].

Mass spectrometry-based technologies, such as gas chromatography time-of-flight mass spectrometry (GC-TOF-MS) and ion molecule reaction mass spectrometry (IMR-MS) have also been employed to investigate breath analysis for IBD [17,18,46]. Smolinska et al. [46] utilised GC-TOF-MS to achieve very promising results for the non-invasive diagnosis of UC [AUC: 0.94, sensitivity: 0.92, specificity: 0.77]. In our previous work, we used field asymmetric ion mobility spectrometry (FAIMS) to separate IBD from controls [AUC: 0.82, sensitivity: 0.74, specificity: 0.75] [8].

While the GC-TOF-MS study by Smolinska et al. was able to exceed the diagnostic performance achieved in this pilot study, the Wolf eNose and G.A.S. BreathSpec GC-IMS are a fraction of the price of GC-TOF-MS and SIFT-MS technologies (10–20% of the cost). Furthermore, the G.A.S. BreathSpec instrument is user-friendly, since it does not require trained operators, and is easy to move when mounted on a trolley. These practical advantages, in combination with chemical identification abilities, provide key advantages for GC-IMS technology in a clinical setting.

In this study, we demonstrated that both eNose and GC-IMS were consistently able to separate those with IBD from healthy control volunteers, regardless of disease activity as reflected by the FCP scores. Additionally, both technologies were able to provide some separation; GC-IMS *p* = 0.026 and eNose *p* = 0.0001, between CD and UC. The results indicate that the G.A.S. BreathSpec GC-IMS is better suited towards distinguishing between IBD and controls, while the Wolf eNose can better separate between CD and UC. These results are expected to be further improved by increasing the number of recruited subjects. The sensor array deployed in the Wolf eNose is focused towards inorganic gases, which most likely accounts for the differences in diagnostic accuracy achieved by the employed technologies. Ammonia, SO_2_ and NO_2_ sensors contributed significantly to the analysis of the Wolf eNose. Ammonia has an established link to IBD breath [16,44,47], since it is one of the intermediaries generated from bacterial fermentation of proteins [48]. UC was associated with higher SO_2_ levels in our study, which has been observed previously [49]. It has been suggested that residential exposures to SO_2_ and NO_2_ may increase the risk of early-onset of CD and UC [50].

Increases in the aforementioned VOCs, butanoic acid and ethanoic acid, contributed significantly to the efficacy of our analysis for G.A.S. BreathSpec GC-IMS. These VOCs have been recently identified as important discriminatory volatile organic metabolites for IBD [51]. Short-chain fatty acids, such as butyric-, propionic- and acetic acids, are produced in the colon by fermentation of fibre. In particular, butanoic acid (also known as butyric acid) is a key component for health in the colon [52] and is the main energy substrate for colonocytes [53]. Butanoic acid has therefore been suggested to play an important role in the prevention and treatment of distal UC [54] and CD [55]. The variations in the identified compounds between IBD subjects and controls may be crucial for diagnostic purposes and need to be further investigated.

Analysis results of possible confounding factors show that BMI and smoking habits have insignificant influence on breath content. In general, the effect of smoking is an obvious factor to influence breath. However, it is worth noting that 19 of the 21 ‘smoker’ subjects consider themselves to be ex-smokers. It is therefore unsurprising that greater differentiation was not possible in this case. Gender showed a more significant influence on breath content than the other two factors [AUC: 0.66, sensitivity: 0.68, specificity: 0.65]. While IBD generally affects men and women equally, some studies from North America show that UC is more common in men than women [56]. The unbalanced gender counts in the UC group (11M: 4F) could therefore be responsible for strengthening the separation between males and females for the confounding factor analysis. In addition to this, gender affects metabolism which can lead to differences in breath content [41]. However, this factor was not significant enough to create two distinct groups or undermine the IBD-related analysis. Age was not considered in the confounder analysis; however, this factor is known to have limited effect on breath content, with some studies showing no statistically significant associations between age and common breath gas metabolites [57]. Moreover, unlike many other diseases, IBD can occur at any age (most likely between 15–35). Age is therefore unlikely to have had a significant effect on the conducted analysis. Lastly, the effect of medication could not be considered in the confounding factor analysis, due to the number and variety of medications and treatments ascribed to each IBD subject. It is possible that the strong results distinguishing between IBD and controls, as well as CD and UC, could be related to the effect of medication; however, the same class of drugs was proportionally present in both IBD groups, so this factor is less likely to be a confounder.

Results from the analysis combining breath analysis with FCP caused specificity, PPV and AUC to reduce, compared to those without FCP. While the differences in mean FCP scores between CD and UC is almost 300 ug/g (414.1–116.9 ug/g), this feature is prone to misclassification because all CD scores fall within the lower range of UC scores. Thus, including FCP with breath analysis does not improve distinction between CD vs. UC within this study. However, as shown in Table 9, FCP did improve separation of CD vs. controls and UC vs. controls. This is likely due to the significantly higher FCP scores in both CD and UC, when compared with the normal reference range of a healthy individual (<50 ug/g) [58]. Nonetheless, this specific application of FCP has little practical or clinical value, since the same conclusions can be derived using FCP scores alone. Moreover, there would be added costs (£18 per test) to conduct FCP tests, alongside breath analysis. These additional costs cannot be justified without a significant improvement in diagnostic performance in distinguishing between CD and UC. GC-IMS and eNose technologies therefore show the greatest potential as non-invasive diagnostic tools for IBD.

## 5. Conclusions

The results from this pilot study confirm the utility of breath VOC analysis to distinguish between IBD and healthy control volunteers, and CD from UC. To the best of our knowledge, this study was the first breath-based investigation of IBD utilising GC-IMS and eNose technology. Both technologies consistently showed the ability to separate those with IBD and controls [AUC ± 95%, sensitivity, specificity], eNose: [0.81 (0.66–0.96), 0.67, 0.89] and GC-IMS: [0.93 (0.85–1.00), 0.87, 0.89]. Furthermore, we were able to separate CD from UC, eNose: [0.88 (0.77–0.98), 0.71, 0.88] and GC-IMS: [0.71 (0.51–0.91), 0.86, 0.62]. The G.A.S. BreathSpec GC-IMS is better suited towards distinguishing between IBD and controls, while the Wolf eNose can better separate between CD and UC. Compound analysis has identified two breath VOCs, which are likely to have a direct link to IBD: butanoic acid and ethanoic acid. These compounds played a crucial role in separating those with IBD from controls. Analysis of possible confounding factors indicate that BMI, smoking habits and gender have insignificant influence on breath content. Wolf eNose analysis was repeated on a reduced dataset, with FCP scores serving as an additional feature. This resulted in a poorer separation of CD and UC, which indicates that the efficacy of breath analysis is reduced, when supplemented with FCP; [0.85 (0.63–1.00), 1.00, 0.67] without FCP, and [0.74 (0.50–0.98), 1.00, 0.56] with FCP. The inclusion of FCP was able to improve diagnostic performance for CD vs. controls and UC vs. controls; however, this application has limited clinical value. Thus, the G.A.S. BreathSpec GC-IMS and Wolf eNose instruments offer the greatest potential as non-invasive, high-throughput, real-time diagnostic and screening tools for IBD, in point-of-care use. Moreover, since breath testing using these technologies could be undertaken during routine consultancy appointments, it has the potential to fundamentally change the current clinical diagnostic and assessment pathways for IBD.

## Figures and Tables

**Table 1:** Demographic data of IBD patients and healthy controls.

Parameter	Crohn’s Disease (*n* = 14)	Ulcerative Colitis (*n* = 16)	Controls (*n* = 9)
Mean age (SD)	46.1 (15.0)	52.8 (19.4)	30.9 (11.5)
Gender ratio M:F	7:7	11:5	4:5
Smoking habits	1 active smoker 7 ex-smokers	1 active smoker 8 ex-smokers	4 ex-smokers
Alcohol—mean units/week (SD)	4.3 (10.0)	8.1 (10.4)	3.7 (4.9)
Mean BMI (SD)	26.1 (8.0)	27.3 (4.9)	27.5 (9.0)
Medication	5-ASA ^1^: 8 AZA ^2^: 4 Anti-TNFα ^3^: 6 PPI ^4^: 4 Salbutamol inhaled: 1 Inhaled steroid: 2	5-ASA ^1^: 13 AZA ^2^: 3 Anti-TNFα ^3^: 3 PPI ^4^: 2 Inhaled steroid: 1	NSAID ^5^: 1 COCP ^6^: 1
Mean HBI/SCAI score (range; SD)	4.5 (0–29; 4.8)	1.5 (0–6; 2.25)	N/A
Disease extent	Ileal disease: 2 Colonic: 3 Ileo-colonic: 9	Pancolitis: 7 Proctitis: 4 Quiescent: 1 Distal colitis: 3 Right-sided: 1	N/A
Mean CRP in mg/L (SD)	7.8 (15.2) 1 unknown	5.3 (4.0) 1 unknown	N/A
Mean FCP in ug/g (SD)	116.9 (112.8) 3 unknown	414.1 (315.5) 7 unknown	N/A

^1^ 5-ASA—aminosalicylate acid; ^2^ AZA—azathioprine; ^3^ Anti-TNFα—anti tumour necrosis factor alpha; ^4^ PPI—proton pump inhibitor; ^5^ NSAID—Nonsteroidal anti-inflammatory drugs; ^6^ COCP—Combined oral contraceptive pill.

**Table 2:** Sensors deployed in Wolf eNose.

Manufacturer	Sensing Method	Target Gases
Alphasense Ltd.	Electro-chemical	O_2_, NH_3_, ETO, SO_2_, O_3_, NO, NO_2_, H_2_S, CO, H_2_
Clairair Ltd.	Infra-red optical	CO_2_, CH_4_
MOCON	Photo-ionization	All

**Table 3:** Summary of confounding factor groups.

Factor	Groups	CD	UC	Controls	Total
BMI	Under- & normal weight	9	5	6	20
	Overweight & obese	5	11	3	19
Smoking	Smokers	8	9	4	21
	Never smokers	6	7	5	18
Gender	Male	7	11	4	22
	Female	7	5	5	17

**Table 4:** IBD analysis: G.A.S. BreathSpec GC-IMS results.

Test	AUC ± 95%	Sensitivity	Specificity	PPV	NPV	*p*-Value
IBD vs. Controls	0.93 (0.85–1.00)	0.87 (0.69–0.96)	0.89 (0.52–1.00)	0.96	0.67	5.1 × 10^−5^
CD vs. UC	0.71 (0.51–0.91)	0.86 (0.57–0.98)	0.62 (0.35–0.85)	0.67	0.83	0.026

**Table 5:** IBD analysis: Wolf eNose results.

Test	AUC ± 95%	Sensitivity	Specificity	PPV	NPV	*p*-Value
IBD vs. Controls	0.81 (0.66–0.96)	0.67 (0.47–0.83)	0.89 (0.52–1.00)	0.95	0.44	0.0019
CD vs. UC	0.88 (0.77–0.98)	0.71 (0.42–0.92)	0.88 (0.62–0.98)	0.83	0.78	0.0001

**Table 6:** Confounding factors: G.A.S. BreathSpec GC-IMS results.

Test	AUC ± 95%	Sensitivity	Specificity	PPV	NPV	*p*-Value
BMI	0.47 (0.28–0.65)	0.63 (0.38–0.84)	0.50 (0.27–0.73)	0.55	0.59	0.642
Smoking	0.58 (0.39–0.76)	0.57 (0.34–0.78)	0.67 (0.41–0.87)	0.67	0.57	0.213
Gender	0.66 (0.48–0.84)	0.68 (0.45–0.86)	0.65 (0.38–0.86)	0.71	0.61	0.229

**Table 7:** Confounding factors: Wolf eNose results.

Test	AUC ± 95%	Sensitivity	Specificity	PPV	NPV	*p*-Value
BMI	0.52 (0.33–0.71)	0.63 (0.38–0.84)	0.55 (0.32–0.77)	0.57	0.61	0.428
Smoking	0.50 (0.32–0.69)	0.48 (0.26–0.70)	0.67 (0.41–0.87)	0.62	0.52	0.494
Gender	0.61 (0.43–0.79)	0.73 (0.50–0.89)	0.47 (0.23–0.72)	0.64	0.57	0.599

**Table 8:** CD vs. UC (without and with FCP): Wolf eNose results.

Test	AUC ± 95%	Sensitivity	Specificity	PPV	NPV	*p*-Value
Breath without FCP	0.85 (0.63–1.00)	1.00 (0.72–1.00)	0.67 (0.30–0.93)	0.79	1.00	0.0037
Breath with FCP	0.74 (0.50–0.98)	1.00 (0.72–1.00)	0.56 (0.21–0.86)	0.73	1.00	0.0311

**Table 9:** CD vs. Controls & UC vs. Controls (without and with FCP): Wolf eNose results.

FCP	Test	AUC ± 95%	Sensitivity	Specificity	PPV	NPV	*p*-Value
Without FCP	CD vs. Controls	0.77 (0.54–0.99)	0.55 (0.23–0.83)	1.00 (0.66–1.00)	1.00	0.64	0.0232
	UC vs. Controls	0.72 (0.45–0.98)	0.89 (0.52–1.00)	0.67 (0.30–0.93)	0.73	0.86	0.0534
With FCP	CD vs. Controls	0.81 (0.61–1.00)	0.73 (0.39–0.94)	0.78 (0.40–0.97)	0.80	0.70	0.0100
	UC vs. Controls	0.90 (0.75–1.00)	1.00 (0.66–1.00)	0.78 (0.40–0.97)	0.82	1.00	0.0023
